# Screening for Pelvic Floor Disorders and Sexual Dysfunction in Postpartum Women: A Retrospective Cohort Study

**DOI:** 10.7759/cureus.65307

**Published:** 2024-07-24

**Authors:** Simone R Fertel, Alyssa Clare, Jean P Tanner, Katie Propst

**Affiliations:** 1 Urogynecology and Reconstructive Pelvic Surgery, USF (University of South Florida) Health, Tampa, USA; 2 Medicine, University of South Florida, Tampa, USA; 3 Public Health, USF (University of South Florida) Health, Tampa, USA

**Keywords:** urogynaecology, preventative health care visits, female sexual health, pelvic floor disorder, gynecology, postpartum care

## Abstract

Objectives

This study assesses the screen rate and prevalence of postpartum pelvic floor disorders and sexual dysfunction (PFDs/SD) within the first year of delivery.

Methods

This is a retrospective review of postpartum women seen in a university clinic who delivered at the associated hospital and had postpartum visits from June 1, 2020, to April 15, 2022. Charts were reviewed from delivery to one year postpartum. Demographic and clinical characteristics were compared between women with and without postpartum screening.

Results

Three hundred thirty-four women met inclusion criteria. Two hundred twenty (65.9%) were screened for PFDs/SD. Compared to women who were not screened, women who were screened were older (32.6 vs 31.3 years, p=0.02). Women with a cesarean delivery (73% vs. 58% vaginal, p=0.004), delivered by an attending or resident (70% vs 60% midwife, p=0.06), first postpartum visit at less than six weeks after delivery (76% vs. 43% 6-12 weeks, p<0.001), and three or more postpartum visits (80% vs. 65% two visits, 50% one visit, p<0.001) were more likely to be screened. In an adjusted model, only timing of the first postpartum visit remained significant. Urinary incontinence and fecal incontinence were the most common PFDs diagnosed. Of the 41 women who had PFDs and/or SD, 31 (75.6%) were referred to pelvic floor physical therapy (PFPT) and/or urogynecology.

Discussion

In this retrospective cohort study, we found a low rate of postpartum screening for PFDs/SDs. This deficiency highlights critical gaps in care for postpartum women.

## Introduction

Postpartum care is just as important as prenatal care, as a woman’s body is changing, and her mood can be affected as well. The recommendation is that all women should have a postpartum visit of some sort with a maternal care provider within three weeks of delivery, followed by a comprehensive postpartum visit within 12 weeks of delivery [1]. However, it is estimated that 40% of women do not receive postpartum care at all [1]. When women are seen at a postpartum visit, there are many issues that need to be discussed including delivery details and any associated complications, recovery, breastfeeding, and socioeconomic factors. Traumatic or complicated deliveries often become the focus of the postpartum visit as well. It is no surprise that pelvic floor disorders can be forgotten at a postpartum visit; however, their incidence is impactful and should be addressed.

Pelvic floor disorders (PFDs) include urinary incontinence (UI), pelvic organ prolapse (POP), and fecal incontinence (FI). In a systematic review of population-based studies, postpartum prevalence of UI was measurable with up to 33% of women affected within the first three months [2]. In terms of prolapse, stage 2 POP was noted in 31% of women after pregnancy and delivery. POP below the hymen was noted in 14%-15% of women after delivery [3]. Long-term effects have also been shown, with a significantly higher odds of POP after spontaneous vaginal delivery compared to a cesarean section in a longitudinal cohort study that reviewed women five to 10 years postpartum. Operative vaginal delivery increased these odds [4]. The Women's Health Initiative study strongly suggested that parity was a significant risk factor for POP as well [5]. FI is just as important as other PFDs. In a prospective cohort study, 8% of primiparous women reported FI when interviewed six months postpartum. This rate was higher in women who experienced an anal sphincter laceration at 17% [6].

Sexual dysfunction can also take a toll on women in the postpartum state. Most women will resume intercourse within the first three months after delivery, and within the year, many will encounter an issue with sexual function [7]. There are many different factors antepartum, intrapartum, and postpartum that can affect sexual health, including medical comorbidities, new-onset preeclampsia, poor obstetrical outcomes, perineal lacerations, and postpartum depression. Anal sphincter lacerations have been shown to have significant impact on sexual function as well [8]. It is important to include sexual dysfunction in the postpartum discussion, as it affects relationships with partners as well as self-image. Although prevalent postpartum, there is very little information on screening for PFDs/SD currently in the literature. This study aims to assess the rate of screening for PFDs/SD in the postpartum period and to review the screening process for these disorders in this setting. This study was previously presented as an oral abstract at the American Urogynecologic Society Pelvic Floor Disorder Week on October 5, 2024.

## Materials and methods

Identification of postpartum women

This is an institutional review board-approved retrospective cohort study. It included postpartum women who were seen in the outpatient setting for a postpartum visit within one year of delivery at the University of South Florida Obstetrics Clinics from June 1, 2020, to April 15, 2022. Women were included if they delivered at the associated hospital and had a postpartum visit at the University of South Florida within the first year of delivery. Women were excluded if they were less than 18 years old, prisoners, were pregnant within one year of delivery, had postpartum care with a urogynecologist, had previous urogynecology visits, had a history of PFDs, or had a first or second trimester loss. The charts of patients who met inclusion criteria were reviewed through the electronic health record (EHR) by two members of the research team to determine if PFDs/SD were addressed at the postpartum visit. Data was collected and managed with REDCap (Research Electronic Data Capture) electronic data capture tools hosted at University of South Florida [9-10].

Data collection

All charts were reviewed to collect data on patient demographics, obstetrical data, screening for PFDs/SD, and management of PFDs/SD, if the screen was positive. Obstetrical data included gestational age at delivery, parity (defined as all deliveries >20 weeks gestation), delivery type, indication for cesarean if applicable, laceration, infant weight, and any hospital complications. Delivery and postpartum clinic providers were also documented as obstetrician attending, obstetrics resident, or midwife. Multiple providers could be selected.

Screening for PFDs/SD was evaluated by reviewing postpartum clinic notes. This included evaluating for pelvic organ prolapse, bladder, or bowel complaints. SD was defined as dysfunction in desire, arousal, orgasm, or sexual pain disorders [11]. If the documentation discussed a PFD/SD in the history of present illness or the assessment and plan or if genitourinary or gastrointestinal was included in the review of systems, this was deemed as a screened patient.

The number of visits within the first postpartum year was documented as well as the timeframe after delivery. If the patient had more than one visit, it was recorded. If the screening was positive, data was collected via the EHR regarding the presence of a disorder (POP, UI, AI, FI, and SD). If multiple diagnoses were documented, all were collected. Treatment was categorized as expectant management, referral to pelvic floor physical therapy (PFPT), pessary discussion or fitting, surgical planning, urogynecology referral, other, or none. Referral to Urogynecology and PFPT was also assessed via EHR notes and orders.

Statistical analysis

The primary objective of this study is to assess the rate of screening for PFDs/SD in the postpartum period. Descriptive characteristics were calculated for demographic and clinical characteristics to describe the study sample and to compare women with and without postpartum screening for PFDs/SD. Continuous variables were reported as mean ± standard deviation, and categorical variables were reported as a number and percentages. Independent-samples t-test for continuous variables and χ^2^ test for categorical variables were used to assess statistically significant differences in clinical and sociodemographic characteristics between patients who did and did not have PFDs/SD. Multivariable log-binomial regression was used to estimate risk ratios (RRs) and 95% confidence intervals (CIs) representing the association between patient characteristics and PFD/SD screening. Only covariates with p-value <0.10 in univariate analyses were included in the adjusted model. All statistical tests were two-sided and declared significant at p<0.05. Statistical analyses were performed with SAS software, version 9.4 (SAS Institute, Inc., Cary, NC).

## Results

During the study period, 3,507 women who received prenatal care at the university clinic delivered at the hospital associated with the university. Three hundred eighty-two (10.9%) patients attended a postpartum visit. Among 382 women who attended a postpartum visit, 334 women met the inclusion criteria. Full patient demographics and delivery characteristics are provided in Table 1. On average, patients were 32.2 ± 4.9 years old. Most patients were white, non-Hispanic (52.7%). The median parity of the patients was 2 (1,2). The median number of postpartum visits was 2 (1, 3), and most patients (272, 81.4%) had at least one visit between six and 12 weeks.

**Table 1:** Demographic and clinical characteristics by screening for pelvic floor disorders or sexual dysfunction ^1^Includes spontaneous and operative vaginal deliveries. ^2^Includes cesareans with and without labor. ^3^Among vaginal deliveries only. Std, standard deviation; PFDs, pelvic floor disorders.

		Were PFDs or sexual dysfunction discussed at any of the postpartum visits?			
Characteristics	All (N=334)	No (N=114)	Yes (N=220)	% PFD discussed	P-value
Age (mean ± std)	32.2 ± 4.9	31.3 ± 4.9	32.6 ± 4.8	NA	0.02
BMI (mean ± std)	29.4 ± 6.8	29.4 ± 7.6	29.4 ± 6.3	NA	0.93
Gestational age weeks (mean ± std)	38.2 ± 2.4	38.1 ± 2.8	38.3 ± 2.2	NA	0.62
Preterm					0.46
Yes	38 (11.4)	15 (13.2)	23 (10.5)	60.5	
Parity					0.34
1	141 (42.2)	48 (42.1)	93 (42.3)	66.0	
2	121 (36.2)	45 (39.5)	76 (34.5)	62.8	
3	53 (15.9)	18 (15.8)	35 (15.9)	66.0	
4+	19 (5.7)	3 (2.6)	16 (7.3)	84.2	
Race/Ethnicity					0.96
White non-His	176 (52.7)	58 (50.9)	118 (53.6)	67.0	
Black non-His	64 (19.2)	21 (18.4)	43 (19.5)	67.2	
Hispanic	52 (15.6)	19 (16.7)	33 (15.0)	63.5	
Asian	15 (4.5)	6 (5.3)	9 (4.1)	60.0	
Other	27 (8.1)	10 (8.8)	17 (7.7)	63.0	
Delivery type					0.004
Vaginal^1^	166 (49.7)	69 (60.5)	97 (44.1)	58.4	
Cesarean^2^	168 (50.3)	45 (39.5)	123 (55.9)	73.2	
Lacerations at time of delivery^3^					0.20
Yes	108 (32.3)	41 (36.0)	67 (30.5)	62.0	
Vaginal wound complication^3^					0.57
Yes	4 (1.2)	1 (0.9)	3 (1.4)	75.0	
Any complications					0.45
Yes	74 (22.2)	28 (24.6)	46 (20.9)	62.2	
Delivery provider					0.06
Attending/resident	208 (62.3)	63 (55.3)	145 (65.9)	69.7	
Midwife only	126 (37.7)	51 (44.7)	75 (34.1)	59.5	
Postpartum care provider					0.53
Attending/resident	233 (69.8)	82 (71.9)	151 (68.6)	64.8	
Midwife only	101 (30.2)	32 (28.1)	69 (31.4)	68.3	
Timing of first postpartum visit					<0.001
<6 weeks	228 (68.3)	54 (47.4)	174 (79.1)	76.3	
6-12 weeks	106 (31.7)	60 (52.6)	46 (20.9)	43.4	
Number of postpartum visits within year of delivery					<0.001
1	89 (26.6)	44 (38.6)	45 (20.5)	50.6	
2	140 (41.9)	49 (43.0)	91 (41.4)	65.0	
3+	105 (31.4)	21 (18.4)	84 (38.2)	80.0	
Did the patient get readmitted within the first six weeks of delivery?					0.43
Yes	16 (4.8)	4 (3.5)	12 (5.5)	75.0	

Among the 334 women in this study, providers discussed at least one PFD/SD with 220 women, making the screening rate for PFDs/SD 65.9%. FI (202, 91.8%) and UI (202, 91.8%) were the most screened disorders. Of those screened, most (179, 81.4%) screened negative. Of the 41 patients who screened positive, the most common PFD documented was UI. Of those who screened positive for PFDs/SD, six (14.6%) were referred to urogynecology, 29 (70.1%) were referred to pelvic floor physical therapy, and 10 (24.4%) proceeded with expectant management.

Compared to women who were not screened, women who were screened were older, more likely to have undergone a cesarean section, delivered by an attending or resident, have their first postpartum visit at less than six weeks following delivery, and attend three or more postpartum visits. Demographic and clinical characteristics by screening for PFDs/SD are given in Table 1.

Age, delivery type, delivery provider, timing of postpartum visits, and number of postpartum visits met the p<0.10 criteria in the univariate analyses and were used in the adjusted model. In the adjusted model only timing of the first postpartum visit remained significant. Women whose first postpartum visit was six to 12 weeks after delivery were 36% less likely to be screened for PFDs/SDs compared to women with their first visit at less than six weeks (RR=0.64; 95% CI: 0.50, 0.81). The results from the log-binomial regression examining the association between patient characteristics and screening for PFDs/SD are displayed in Table 2.

**Table 2:** Association between patient characteristics and screening for pelvic floor disorders or sexual dysfunction RR, risk ratio; CI, confidence interval.

Characteristics	Unadjusted RR (95% CI)	Adjusted RR (95% CI)
Age	1.02 (1.00-1.03)	1.01 (1.00-1.03)
Delivery type		
Vaginal	Ref.	Ref.
Cesarean	1.25 (1.07-1.47)	0.97 (0.74-1.29)
Delivery provider		
Attending/resident	Ref.	Ref.
Midwife only	0.85 (0.72-1.01)	1.03 (0.78-1.35)
Timing of first postpartum visit		
<6 weeks	Ref.	Ref.
6-12 weeks	0.57 (0.45-0.72)	0.64 (0.50-0.81)
Number of postpartum visits within year of delivery		
1	Ref.	Ref.
2	1.29 (1.01-1.63)	0.97 (0.77-1.21)
3+	1.58 (1.26-1.98)	1.16 (0.94-1.44)

## Discussion

Principal findings

In this retrospective cohort study, we found one-third of postpartum patients were not screened for PFDs/SD at any of their postpartum visits. Several factors were found to be associated with a greater likelihood of screening: age, delivery by cesarean section, delivery by attending or resident, three or more postpartum visits, and a first visit less than six weeks postpartum. However, only timing of the first postpartum visit was significant in an adjusted model. Most women with positive screening were referred to urogynecology or pelvic floor physical therapy. These findings allude to a gap in screening postpartum women for PFDs/SD. On a larger scale, the estimated return rate to clinic is very low, which estimates about 10% postpartum follow-up.

Clinical implications

Although women may want to discuss PFDs/SD in a clinic visit, it may be difficult for a patient to raise the topic without being prompted. SD is thought to be under-reported due to uneasiness to discuss the subject and also the lack of training providers have on sexual education [12]. Women may not initiate the discussion with the provider because they may feel embarrassed or ashamed about leakage [13]. Studies have shown that women are interested in learning about bladder health but do not know much about the bladder due to lack of educational resources and not knowing where to obtain reliable information [14]. This phenomenon most likely applies to the other PFDs/SD as well. Besides feeling shame in their symptoms, it is also possible women feel as though PFDs/SD postpartum is considered “normal” and not report these symptoms to providers without being specifically asked. Therefore, documentation of PFDS/SD may not reveal true prevalence. This could account for the inadequate screening rate in our study sample and the low number of positive screens of those who were asked about PFDs/SD. Given the impact and prevalence of PFDs/SD, it is the responsibility of the postpartum care providers to address and educate patients on these matters.

Validated questionnaires have shown reliable findings compared to in-person discussions or patient-reported event logs [15]. Implementation of validated questionnaires for PFDs/SD in the postpartum period could reveal the true prevalence of these disorders and minimize barriers. They could also help prompt providers to inquire further about abnormal answers to the questionnaire and discuss bothersome PFDs/SD in detail and refer to an advanced provider.

This study also revealed a deficiency in postpartum care after delivery. The women in this study are not an accurate representation of all postpartum women because there was only about a 10.9% postpartum return rate. This finding was quite surprising, as the postpartum return rate was lower than what is cited in the literature. It has been estimated that 40% of women do not have postpartum care [1]. It is possible that the low postpartum attendance rate for this cohort and for postpartum patients on a larger scale is due to the lack of communication about the follow-up visit when leaving the hospital after delivery. It is imperative to have a clinic date set for postpartum patients prior to discharge from the hospital; otherwise, contact may not be attainable. There also may be an aspect of financial and socioeconomic barriers that may affect attendance to these visits, and this may be the case in our cohort given the demographics of the participants in our study (predominantly white and non-Hispanic). The demographics of this cohort do not match the demographics of our patient population as whole in our area. When asked about why postpartum care was not scheduled, women in one study discussed scheduling difficulties due to work and school, inadequate childcare, and the overall struggles with having a newborn [16]. Having a patient-centered approach to postpartum care could help tailor these visits and increase attendance rates.

The inclusion period for this study was during the global pandemic of the coronavirus disease 2019 (COVID-19), which can contribute to the low rate of postpartum care. Many of the charts reviewed in this study were telehealth clinic visits, and postpartum attendance rate has not seen a difference with this implementation during coronavirus disease of 2019 (COVID-19) [17]. A noted advantage for telehealth visits in the psychiatry realm was the increased personal safety for patients who were at risk for violence and behavioral dysregulation [18]. Anecdotally, virtual clinic would allow the patient and provider to discuss more symptoms in lieu of a physical exam and could also increase the privacy needed to discuss sensitive topics.

Study strengths and limitations

Strengths of this study include data collection from a robust EHR linking both inpatient and outpatient records and systematic data collection by well-trained study personnel. This study is limited by its retrospective nature. It is possible that PFDs/SD were discussed but not documented in the medical record. Since genitourinary or gastrointestinal recorded in review of symptoms counted as a screen, it is also possible that the PFD/SDs screening number is actually lower than reported. This would explain the higher likelihood of screening in patients with cesarean sections.

Studies have reported increased adherence to postpartum counseling using an EHR template [19]. The prevalence of PFDs/SDs for this cohort may not be generalizable given these limitations and because of the homogeneity of participants. Billing and coding for visits after delivery could have affected the charts that were collected for this study as well. If a visit was not billed or coded for a postpartum state or visit, the patient was not included in the study.

Future aims

Future goals should focus on postpartum PFDs/SD since these are symptoms that are often overlooked at a postpartum visit. Providers should be trained on screening and treatment of these disorders to provide a comprehensive postpartum visit. It is possible that including a section on PFDs/SD on a postpartum template in the EHR can increase awareness and documentation for providers. Barriers to scheduling postpartum care appointments should be minimized and their importance should be emphasized during antenatal care. Postpartum care should also be a patient-centered discussion, and there may need to be unconventional postpartum visits to accommodate women.

## Conclusions

In this retrospective cohort study, we found a low rate of postpartum follow-up and screening for PFDs/SDs. These deficiencies highlight critical gaps in care for postpartum women. Future goals should include medical education on screening in order to increase awareness of and educate women on PFDs/SD, as well as implementation of templated EHR notes that include this screening. There also needs to be an emphasis on postpartum care in order to increase the follow-up rate and improve these outcomes.
